# Beneficial Effects of Melatonin Combined with Exercise on Endogenous Neural Stem/Progenitor Cells Proliferation after Spinal Cord Injury

**DOI:** 10.3390/ijms15022207

**Published:** 2014-01-30

**Authors:** Youngjeon Lee, Seunghoon Lee, Sang-Rae Lee, Kanghui Park, Yunkyung Hong, Minkyung Lee, Sookyoung Park, Yunho Jin, Kyu-Tae Chang, Yonggeun Hong

**Affiliations:** 1 Department of Rehabilitation Science, Graduate School of Inje University, 197 Inje-ro, Gimhae, Gyeong-nam 621-749, Korea; E-Mails: neurosci@kribb.re.kr (Y.L.); stormyboy@nate.com (S.L.); jspt95@hanmail.net (K.P.); dangmoo777@naver.com (Y.H.); saesee2000@hanmail.net (M.L.); 2 National Primate Research Center (NPRC), Korea Research Institute of Bioscience and Biotechnology (KRIBB), 30 Yeongudanji-ro, Ochang, Chung-buk 363-883, Korea; E-Mail: srlee@kribb.re.kr; 3 Cardiovascular & Metabolic Disease Center, College of Biomedical Science & Engineering, Inje University, 197 Inje-ro, Gimhae, Gyeong-nam 621-749, Korea; E-Mail: charm-soo@hanmail.net; 4 Department of Physical Therapy, College of Biomedical Science & Engineering, 197 Inje-ro, Gimhae, Gyeong-nam 621-749, Korea; 5 Ubiquitous Healthcare Research Center, 197 Inje-ro, Gimhae, Gyeong-nam 621-749, Korea

**Keywords:** spinal cord injury, melatonin, functional recovery, endogenous neural stem/progenitor, therapeutic exercise

## Abstract

Endogenous neural stem/progenitor cells (eNSPCs) proliferate and differentiate into neurons and glial cells after spinal cord injury (SCI). We have previously shown that melatonin (MT) plus exercise (Ex) had a synergistic effect on functional recovery after SCI. Thus, we hypothesized that combined therapy including melatonin and exercise might exert a beneficial effect on eNSPCs after SCI. Melatonin was administered twice a day and exercise was performed on a treadmill for 15 min, six days per week for 3 weeks after SCI. Immunohistochemistry and RT-PCR analysis were used to determine cell population for late response, in conjunction with histological examination and motor function test. There was marked improvement in hindlimb function in SCI+MT+Ex group at day 14 and 21 after injury, as documented by the reduced size of the spinal lesion and a higher density of dendritic spines and axons; such functional improvements were associated with increased numbers of BrdU-positive cells. Furthermore, MAP2 was increased in the injured thoracic segment, while GFAP was increased in the cervical segment, along with elevated numbers of BrdU-positive nestin-expressing eNSPCs in the SCI+MT+Ex group. The dendritic spine density was augmented markedly in SCI+MT and SCI+MT+Ex groups. These results suggest a synergistic effect of SCI+MT+Ex might create a microenvironment to facilitate proliferation of eNSPCs to effectively replace injured cells and to improve regeneration in SCI.

## 1. Introduction

The stem cell field may serve as the glue that bonds and focuses many multidisciplinary approaches for spinal cord repair [1]. Endogenous neural stem/progenitor cells (eNSPCs) are found in the ependymal regions lining the central canal in the spinal cord [2]. They are capable of proliferation, migration, and maturation into oligodendrocytes, astrocytes, and neurons [3,4], and play an important role in reorganization after spinal cord injury (SCI) [5]. In addition to their plasticity in cell replacement, eNSPCs avoid problems of tumor formation, immune rejection response, and ethical concerns, which are significant challenges for exogenous stem cell transplantation-based approaches [6]. Several therapeutic strategies to activate eNSPCs have been investigated, including administration of granulocyte-macrophage colony stimulating factor (GM-SCF) [7], leukemia inhibitory factor (LIF) [8], and a combination of neuronal transcription factor overexpression and growth factor treatment [9]. Thus, identification of regulating factors to activate eNSPCs could provide a new therapeutic strategy after SCI. However, extrinsic factors to efficiently stimulate eNSPCs after SCI are not fully understood.

It has been reported that eNSPCs of the intact adult spinal cord are quiescent and present in the ependymal layer or the sub-ependymal zone of the central canal [10,11]. These cells can be isolated and expanded for transplantation from spinal cord [12,13]. In response to injury, eNSPCs of the spinal cord have been shown to proliferate and mature into oligodendrocytes or astrocytes after five weeks, using BrdU labeling [14,15]. Additionally, they migrate towards the lesion site, contributing to glial scar formation [16].

Melatonin (MT, *N*-acetyl-5-methoxytryptamine) is a neurohormone that is synthesized and secreted from the pineal gland, as well as in peripheral tissues, in a circadian fashion [17]. In addition to physiological roles of melatonin, such as in circadian regulation, recent studies have shown that melatonin can enhance adult stem cell viability, proliferation, and differentiation, including that of mesenchymal stem cells into osteoblasts [18,19] and eNSPCs into neurons [20–23], suggesting the application of melatonin in regulating neurogenesis of eNSPCs in the central nervous system, including the spinal cord. Additionally, melatonin stimulates proliferation of eNSPCs and promotes neuronal differentiation under hypoxic condition *in vitro* [24]. However, to date, there has been no reported study evaluating melatonin treatment of eNSPCs after SCI.

Physical exercise has been demonstrated to affect the regulation of neurogenesis not only in the hippocampus [25,26] but also in the spinal cord after SCI [10]. A recent study showed that voluntary exercise affected both neurogenesis and oligodendrogenesis in the thoracic segment of the intact adult spinal cord [27].

In patients with SCI, physical exercise intervention should be selected carefully and might even need to be modified for a pre-clinical study, because exercise training alone in the early phase had few beneficial effects on improved motor function [28,29]. Previously,we reported that SCI+MT+Ex group had a synergistic effect on functional recovery, as the Basso, Beattie and Bresnahan (BBB) scores increased more rapidly and were significantly higher than in the SCI+Ex and non-SCI control (Con) group until day 28 [28,30]. In addition, the SCI+MT+Ex group had significantly reduced iNOS mRNA levels and more motor neurons in the ventral horn, as compared to the Con and SCI+Ex groups, suggesting that a novel therapeutic strategy using melatonin combination with exercise reduced the side effects related to exercise-induced fatigue, impairment, and injury-induced secondary damage [28,30]. In this respect, we further questioned the mechanisms underlying the beneficial effects of melatonin combined with exercise in SCI. This study was undertaken to examine the effect of the SCI+MT+Ex regime on eNSPCs in the adult rat spinal cord after injury, to elucidate whether proliferating eNSPCs are being stimulated, and what cell type is being replaced.

## 2. Results

### 2.1. The Synergistic Effects of Melatonin and Treadmill Exercise Dual Treatment

**Figure 1.**
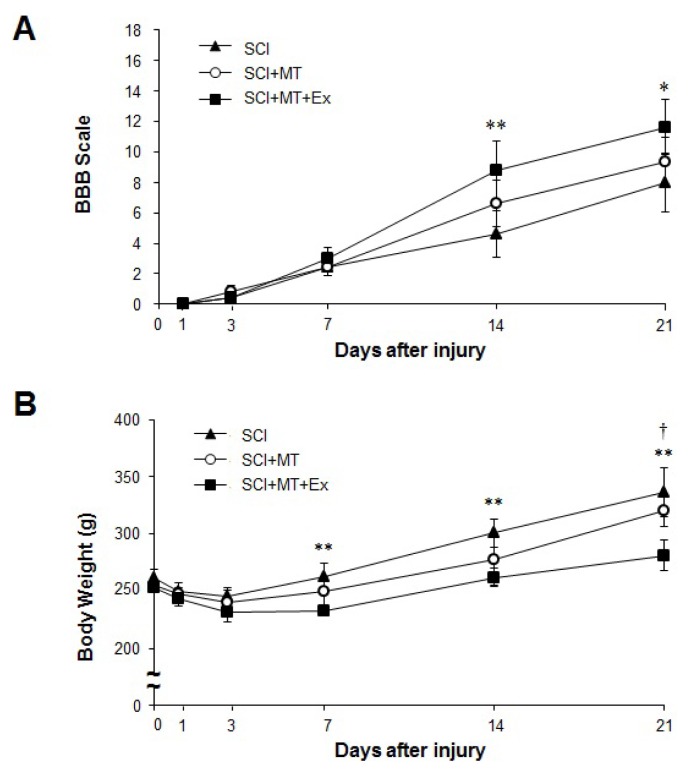
Effect of therapeutic interventions of melatonin and melatonin combined exercise on motor function recovery after spinal cord injury. (**A**) Recovery of motor function and change of body weight during 21 days after SCI. Values of the BBB rating scale are presented. Rats were treated with saline (SCI) or melatonin (SCI+MT, SCI+MT+Ex) from day 1 after injury. Three days after injury, treadmill exercise was combined with melatonin (SCI+MT+Ex). SCI+MT+Ex group demonstrated significant improvement at day 14 and 21 after injury, compared with SCI (* *p* < 0.05, ** *p* < 0.01). (**B**) SCI+MT+Ex induced less gain of body weight compared with SCI. There were significant differences between SCI+MT+Ex and SCI group at day 7, 14 and 21, respectively (** *p* < 0.01). Moreover, the body weight of SCI+MT+Ex was lower than SCI+MT at day 21 († *p* < 0.05). Statistical significance test was done by one-way ANOVA. Data are shown as the mean ± SEM.

To assess behavioral outcome after SCI, we used the BBB scale in which a score of 0 indicates complete paralysis of hind limb movement and 21 indicates intact locomotor performance (Figure 1A). Rats in all experimental groups showed flaccid paralysis with little or no hind limb movement throughout the first week after SCI. Then, all groups improved, with progressive recovery of hind limb movement until 21 days. A statistical analysis of the BBB scores at different time points is shown in Figure 1A. In the acute phase, there was no significant difference in BBB scores between the groups, from 1 to 7 days after injury. By days 14 and 21 after the injury, the SCI+MT+Ex group had a higher motor score (8.8 ± 1.9, 11.6 ± 1.8, respectively) than the SCI group (4.6 ± 1.5, 7.8 ± 1.9, respectively); there was a statistically significant difference in BBB scores between SCI and SCI+MT+Ex (*p* < 0.01, *p* < 0.05, respectively). In the BBB score, 9 points indicate hind limb weight–bearing ability; differences of 8 or 9 points are clearly detectable, and recovery of the capacity to use the hind limb was obvious, whether weight-bearing or not.

To assess physiological changes, body weight was measured and compared according to time-course in the experimental groups. In all groups, body weight decreased during the three days following SCI. Subsequently, body weight increased continuously in all groups (Figure 1B). The body weight of the SCI group increased more than that of the SCI+MT and SCI+MT+Ex groups at day 21 after SCI.

These results indicate a synergistic interaction between physical exercise and exogenous melatonin administration, because melatonin treatment combined with exercise significantly improved functional recovery, as compared with SCI. Although melatonin treatment without exercise also resulted in a slight increase in motor recovery relative to the SCI control, there was no statistically significant difference. Because improvement of hind limb function could increase physical activity in daily living, the SCI+MT+Ex group gained less body weight.

### 2.2. Reconstruction of Spinal Cord Lesion through Melatonin and Treadmill Exercise Dual Treatment

**Figure 2.**
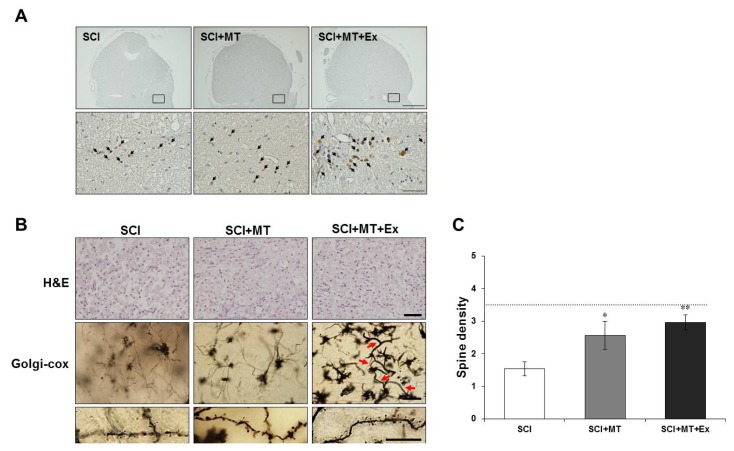
Effect of therapeutic interventions of melatonin and melatonin combined exercise on damaged architectures after spinal cord injury. (**A**) Melatonin combined with exercise increased the BrdU-positive cells, but melatonin did not show a synergistic effect on dividing cell proliferation at day 21 after SCI. Representative cross sections taken 1 mm rostral to the epicenter from SCI, SCI+MT, and SCI+MT+Ex rats, respectively. Immunohistochemistry was performed for BrdU at day 21 after injury. Nuclei were counterstained with hematoxylin. Scale bar = 0.5 mm (upper panel), 50 μm (lower panel). (**B**) Spinal cord tissues at day 21 after injury were observed by H & E staining (upper panel) and Golgi-cox staining (middle and lower panel) at high magnification within the cyst in all groups. Scale bar = 50 μm. (**C**) Quantification of spine density (number of spines/μm) in all groups. The gray dotted line indicates the dendritic spine density for the non-SCI control. Comparisons were made using one-way (ANOVA) with SPSS software and the *post hoc* Tukey’s tests. Data are expressed as the mean ± SEM. (* *p* < 0.05, ** *p* < 0.01 *vs.* SCI).

To evaluate whether loss of motor function, measured using the BBB hind limb locomotor rating scale score was associated with histological damage to the spinal cord, paraffin-embedded longitudinal sections of spinal cord were prepared, and hematoxylin and eosin (H & E) and Golgi-cox staining were evaluated at day 21 after injury (Figure 2B). The severity of the trauma at the level of T9-11 (the lesion area) was assessed by the presence of cysts and edema as well as alterations in the white matter. We combined all 40× magnified light microscope images of the T9-11 segments for histological analysis (Figure 2A).

Significant damage to the spinal cord was observed in the tissue from the SCI rats compared with the non-SCI control rats (Figure 2B). Notably, a significant cell population inside the cyst was observed in all groups at day 21 after injury (Figure 2B, upper panel). Alterations in the morphology of neurons and glia cells were observed by Golgi-cox staining that selectively impregnates single neurons with silver chromate (Figure 2B, middle panel). Significant alterations in morphology and cell loss were observed in the spinal cord tissue from SCI rats when compared with normal control. Moreover, marked neurite outgrowth and newly generated vessels (red arrow) were observed in rats treated with melatonin and exercise at day 21 after injury (Figure 2B, middle panel).Measuring dendritic spines, SCI+MT and SCI+MT+Ex groups significantly increased the number of spines compared with SCI group (SCI+MT: *p* < 0.05, SCI+MT+Ex: *p* < 0.01) (Figure 2B, lower panel).

### 2.3. Melatonin Plus Treadmill Exercise-Induced New Differentiation for Reorganization of Spinal Cord Structure

**Figure 3.**
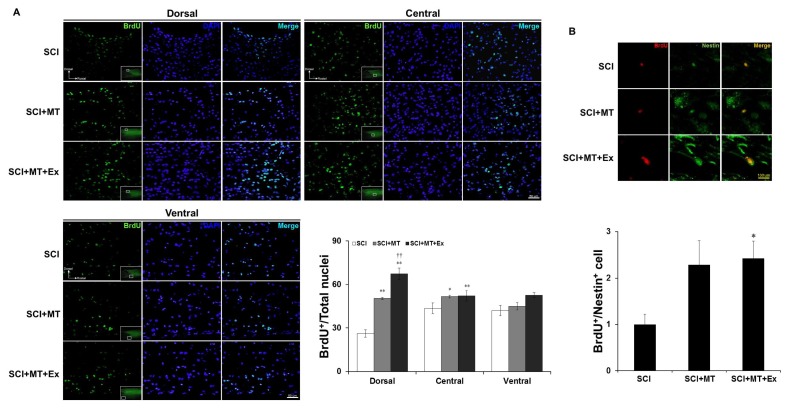
Effects of melatonin and melatonin combined with therapeutic exercise on proliferation of neural stem/progenitor cells after SCI. (**A**) BrdU-positive proliferative cells in the dorsal, central, and ventral site of rostral perilesion at day 21 after SCI.Parasagittal section of rat spinal cord labeled with BrdU (green) and DAPI (blue) in SCI, SCI+MT, and SCI+MT+Ex groups at day 21 after spinal cord injury. BrdU-labeled cells are an index of newly born dividing cells because BrdU is incorporated into replicating DNA; DAPI-labeled nuclei are an index of total cells because they bind to double-stranded DNA. In the merged images, colocalization of BrdU and DAPI is labeled in sky-blue.(**B**) Melatonin combined with exercise doubled the proportion of BrdU-positive cells that were also nestin-positive endogenous neural stem/progenitors at day 21 after injury. BrdU-positive and nestin-positive cell morphology in the 1 mm rostral perilesion site in longitudinal sections at day 21 after spinal cord injury. BrdU staining (red), nestin staining (green), and their co-localization (yellow). Comparisons were conducted by one-way ANOVA using the SPSS software and *post hoc* Tukey’s tests. Data are expressed as mean ± SEM. (*p < 0.05, ** *p* < 0.01 *vs.* SCI; †† *p* < 0.05 *vs.* SCI+MT).

To determine whether proliferative activity derived from the endogenous neural stem/progenitor cells is required for the observed synergistic effect of melatonin and exercise, BrdU was administered for two consecutive days at 19 and 20 days after injury. Subsequently, a BrdU incorporation assay was performed at day 21. Because BrdU-labeled cells are an index of newly born dividing cells, as previously reported [23,24], labeling cells in S-phase, we examined cross sections 1 mm rostral to the epicenter at day 21 after injury, and compared the cells immunostained with BrdU between the groups. BrdU-positive cells were observed distributed throughout the rostral perilesion site, including dorsal, central, and ventral regions. This observation was further confirmed with the quantification of BrdU-positive cell proportion in the total of DAPI-positive nuclei by immunofluorescence microscopy. BrdU-positive cells were abundant in the area of neuro-inflammation in the dorsal column in all groups, reflecting the high proliferative activity of cells in the compression-induced lesion site. As shown at Figure 3A, SCI+MT group significantly augmented the number of BrdU-positive cells in the dorsal and central region compared with SCI (*p* < 0.01). Interestingly, SCI+MT+Ex group showed the highest number of BrdU-positive cells in the dorsal and central region at day 21 after injury cord (*p* < 0.01).

Because recent studies have reported that BrdU-positive cells consist of other cell types, including mature oligodendrocytes and astrocytes, as well as oligodendrocyte precursors [24], we performed double labeling of cells that incorporated BrdU using antibodies against nestin, as another marker of neural progenitor cells [8], to more sensitively detect neural stem/progenitor cells (Figure 3B). Notably, significantly increased numbers of BrdU/nestin double positive cells were found in the 1 mm rostral perilesion site on longitudinal sections from SCI+MT+Ex group at day 21 after spinal cord injury, indicating a synergistic effect of melatonin and exercise on behavioral recovery associated with a nestin-positive neural stem cell population.

**Figure 4.**
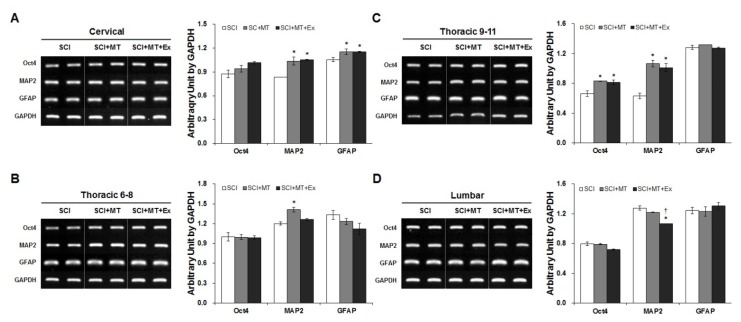
Agonistic effect of therapeutic exercise on endogenous neural stem/progenitor cells proliferation induced by melatonin after SCI. (**A**–**D; left panels**) Effect of melatonin and melatonin combined with exercise on mRNA expression of neuronal, glial, and stem/progenitor markers at day 21 after injury. *Oct4*, *MAP2*, and *GFAP* expression were evaluated by RT-PCR analysis in the four segments of spinal cord. *GAPDH* was used as a loading control. (**A**–**D; right panels**) Densitometry analysis of band intensity in RT-PCR. Image analysis was carried out using the Image J software Comparisons between experimental groups were conducted by one-way ANOVA using the SPSS software and *post hoc* Tukey’s tests. Data are expressed as mean ± SEM. (* *p* < 0.05 *vs.* SCI; † *p* < 0.05 *vs.* SCI+MT).

To further investigate whether the eNSPCs cells may develop into functional neurons and glia, or remain as dividing cells at this chronic phase, RT-PCR was performed to compare marker expression at the mRNA level (Figure 4A–D). At 21 days after SCI, the expression level of *Oct4*, a specific marker of endogenous stem cell-like cells, was only enhanced on thoracic 9–11 regions in SCI+MT and SCI+MT+Ex groups (*p* < 0.05) (Figure 4C). Both *MAP2* and *GFAP*, or *MAP2* levels alone, were increased on cervical, and thoracic 9–11 (lesion) segments, respectively, in the SCI+MT and SCI+MT+Ex groups (*p* < 0.05). By contrast, MAP2 was increased only in the SCI+MT group at the thoracic segments 6–8, rostral to the injured spinal cord (*p* < 0.05).

## 3. Discussion

For the past couple of decades, clinicians have inspected stem cell therapy with a mixture of expectation and skepticism. Therapies for spinal cord injury have been promised almost since the dawning of the stem cell approaches. Although stem cell therapy, like many in the field of spinal cord injury, is not without flaws, it provides an opportunity to coach clinicians about why research in spinal cord injury is so challenging [1]. Therefore, we investigated new strategies using pharmacological and physiological intervention on SCI. This study demonstrates a synergistic effect between melatonin treatment and physical exercise in a rat spinal cord injury model with behavioral improvement, histological recovery, and increased BrdU/nestin double positive endogenous eNSPCs numbers. The functional contributions of the newly forming neural cells derived from the BrdU/nestin double positive cells have not yet been characterized. We suggest nestin-positive neural stem cells could be induced by dual treatment, and that they may contribute to the replacement of cells lost, and differentiation of neurons and glia, which are more effectively integrated eventually into neural circuits. Thus, we suppose that melatonin treatment combined with exercise contributed to functional recovery and pathophysiological changes via an increase in nestin-positive eNSPCs.

Contusion-induced SCI in rats at the T10 level resulted in severe trauma, characterized by edema, cysts, and loss of white matter, primarily in the dorsal column. This histological damage was associated with a loss of motor function. Melatonin and melatonin plus exercise promptly induced spherical cells at the early phase, which were maintained until the chronic phase. We assumed that proliferative activity was exerted by endogenous neural stem/progenitor cells; they were analyzed by a BrdU immunoreactive assay and other stem cell marker mRNA expression, such as *Oct4*. Cell proliferation activity was more prominent in the melatonin-treated group at the early phase than in the dual treatment group. In chronic phase, however, there was no significant difference between the groups. Thus, functional recovery is not consistent with a simple quantitative increase in BrdU-positive endogenous proliferative activity, but involves qualitative reconstruction, including neural connections and synaptic signal transduction, and this may be associated with nestin-expressing cells. Our data provide evidence that treadmill exercise with melatonin treatment promotes the generation of cells that are the nestin-labeled cells in the adult spinal cord. We showed that dual treatment had a synergistic effect on nestin expression in the rostral perilesion site, suggesting that nestin may be related to functional recovery. Using Golgi-cox analysis, we found that SCI+MT+Ex group induced an increase in dendritic spine density. A dendritic spine is a small membranous protrusion from the dendrite of a neuron that typically receives input from a single synapse of an axon [31]. An increase in dendritic spine density indicates augmented transmission of nerve impulses from the brain to peripheral nerves [32]. eNSPC-derived neural cells might be locally reorganized neural circuits in the spinal cord. Consistent with this, previous studies reported that voluntary exercise induced nestin expression with improved motor function [25,33]. In this study, there is a limitation in not comparing the effects of exercise alone and exercise combined with melatonin treatment. However, we suggest that the effect of exercise combined with melatonin treatment may be similar to the effect of exercise alone, or better, because melatonin has been reported to be a short-lived hormone with negligible side effects that is rapidly degraded by and eliminated from the body; in addition, there were no adverse effects in a human study [34,35].

This report provides the first demonstration that melatonin can enhance endogenous adult stem cell proliferation in spinal cord injury. It is not yet clear which molecular mechanism(s) underlie the effect of melatonin on adult neural stem cells. However, it seems possible that some of its effects are mediated via the melatonin receptors. MT_1_ and MT_2_ melatonin receptors are present in the dorsal and ventral horn of the spinal cord [36] as well as in neural precursor cells [20]. These receptors are activated by melatonin and mediate cell metabolism and proliferation via activation of insulin and the IGF-1 signaling cascade, which activate the PI3K/AKT and MEK/ERK pathways, respectively [37]. They also modulate cell survival and neuronal differentiation [20]. Consistent with this, we found that the groups given exogenous melatonin supplements (SCI+MT and SCI+MT+Ex) had significantly more proliferating cells and higher expression of *MAP2*, a marker for mature neurons, in the lesion at the chronic phase, but not in the early phase, suggesting that neuronal differentiation is not complete by day seven after injury (data not shown). Recently, Du *et al*. [38] stated that neural stem cells transplantation promoted differentiation into *MAP2* and *PSD-95* in perilesional sites. This contributes to new synaptogenesis between the upper region and injured sites [38]. Therefore, our data show the reorganization of the neural structure following the combination of supplemental melatonin and exercise.

Our data also provide evidence for a synergistic effect of melatonin and exercise on functional recovery. This synergistic effect could be mediated by a reduction in secondary damage. We have previously reported that this dual treatment caused a decreased level of iNOS expression, which aggravates neuronal damage after spinal cord injury [28]. Exercise has been reported to increase many kinds of neurotrophic factors, such as BDNF, EGF, and FGF-2, which are known to be involved in regulating proliferation, differentiation, and survival of adult neural stem/progenitor cells in the brain [39–41]. Melatonin has been reported to have an anti-nociceptive effect in SCI [36]. These studies suggest that melatonin administration enhanced treadmill-induced movement for conducting physical exercise as a result of pain modulation. Furthermore, endogenous adult neural stem/progenitor cells secrete various neurotrophic factors constitutively, including BDNF [42]. Here, we showed that the melatonin-treated groups induced increases in eNSPCs around the lesion; this might lead to promoting trophic effects of exercise.

In conclusion, the current study suggests that exogenous melatonin administration combined with physical exercise increases histological and behavioral recovery. Additionally, this dual treatment appears to increase nestin-positive eNSPCs, driving effective reconstructed neuronal differentiation. Our findings suggest that melatonin may have therapeutic potential because it contributes to activating adult neural stem cells; at this time, conducting exercise simultaneously may help to enhance the intrinsic potential for recovery from spinal cord injury.

## 4. Experimental Section

### 4.1. Experimental Animals

**Figure 5.**
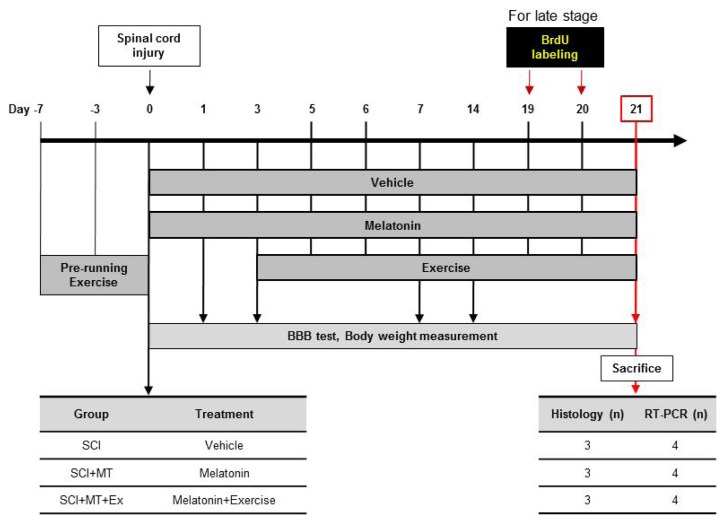
Schematic representation of experimental design.

All procedures in this study were conducted in accordance with a protocol approved by the Ethics Committee for Animal Experiments at Inje University (Approval No. 2010-24). In total, 40 male 8-week-old Sprague-Dawley rats (250–270 g) underwent SCI surgery. The 19 that developed health problems or died were excluded from this research. The remaining 21 rats were used for the BBB behavioral test. During the experiments, all rats were housed in standard plastic cages (20 × 10 × 10 inches) in a controlled temperature (22 ± 2 °C), relative humidity (55% ± 5%), and light/dark conditions (12/12-h light/dark cycle) room before use. Food and water were available *ad libitum*. Rats were randomly divided into three groups, SCI, SCI+MT, and SCI+MT+Ex, as indicated in Figure 5.

### 4.2. Spinal Cord Injury Animal Model

Rats were anesthetized with an intraperitoneal injection of mixture of tiletamine/zolazepam(40 mg/kg of body weight, Zoletil; Virbac, Carros, France) and xylazine (10 mg/kg body weight, Rompun; Bayer Healthcare, Seoul, Korea). A contusive injury was performed as described previously [28]. Briefly, after laminectomy of T9, the T10 level was exposed with the dura matter remaining intact. Spinal cord contusion was induced by a modified NYU weight-drop device [42,43]. A 10 g weight rod was allowed to drop from a height of 25 mm onto the dorsal surface of the dura matter.

### 4.3. Melatonin Treatment

Melatonin (Sigma, St. Louis, MO, USA) was dissolved in absolute ethanol and diluted in saline solution (final concentration of ethanol was 0.5%) [28]. Rats were injected subcutaneously with melatonin at doses of 10 mg/kg body weight [28]. Starting one day after the operation, rats (SCI+MT, SCI+MT+Ex) were injected twice a day (07:00, 19:00) until sacrifice.

### 4.4. Treadmill Exercise

Rats (SCI+MT+Ex) were trained to walk quadrupedally on the treadmill starting three days after the operation. Training was performed twice a day (17:00, 22:00) for 15 min, at a speed of 10.5 m/min, six days per week [28,44]. If the rats were not capable of plantar stepping, body weight support was provided by manually lifting to partially unload the hindlimbs. When no stepping of the hind limbs occurred in response to the moving treadmill and the stepping of the forelimbs, it was elicited by manual stimulation of the perineum. The grade of support was adjusted to make sure that the hind limbs of the animals did not collapse and was gradually removed as motor function improved. Additionally, during the first week of training, when all group rats showed paraplegia, assistance was provided, placing the rat hindlimbs in plantar-stepping position during training.

### 4.5. Bromodeoxyuridine (BrdU) Injection

Proliferating cells were labeled with BrdU (Sigma, St. Louis, MO, USA), with a 50 mg/kg body weight intraperitoneal injection once daily over two days before the end of the experiment. BrdU was dissolved in DMSO with heating, and the stock concentration was 100 mg/mL.BrdU labels new cells by incorporation into replicating DNA [43].

### 4.6. Assessment of Motor Function (the BBB Open Field LocomotionTest)

In all groups, locomotor behavior was assessed using the Basso, Beattie, and Bresnahan (BBB) locomotor recovery scale, which consist of a 21-point scale [44,45]. This scale was assessed with observation of hind limb movements, stepping, and coordination in an open field. Spinal cord-injured animals were tested on postoperative days 1, 3, 7, 14, and 21, as described previously [28].

### 4.7. RNA Isolation and RT-PCR Analysis

Rats were anesthetized as described above. Then, each segment of spinal cord was removed from an injured area depending on the level, as follows: cervical (C5–7), mid-thoracic (T6–8), lower thoracic (T9–11), and lumbar (L1–2). Spinal cord tissue was homogenized with 1 mL of Tri-reagent (Sigma, St. Louis, MO, USA) to prepare total RNA. The RNA was reverse-transcribed with oligo(dT)_15_ primers (Promega, Fitchburg, WI, USA) and SuperScript II reverse transcriptase (Invitrogen, Carlsbad, CA, USA); this reaction mix served as a template for the polymerase chain reaction (PCR) as previous reported [34]. Primer sets are shown in Table 1.

### 4.8. Tissue Processing and Immunohistochemistry

For the BBB behavioral test, 21 rats were anesthetized as described above and perfused intracardially with 4% paraformaldehyde in 0.1 M phosphate buffer, pH 7.4, for 15 min. After fixation, post-fixation was performed overnight in 4% paraformaldehyde. For fluorescence immunostaining, non-specific labeling was blocked with 0.1% BSA in 0.1% Triton X-100/PBS for 60 min. The following primary antibodies were used and incubated with the tissue overnight at 4 °C: mouse monoclonal anti-BrdU (1:100, Santa Cruz Biotechnology Inc., Santa Cruz, CA, USA), rabbit polyclonal anti-nestin (1:200, Santa Cruz Biotechnology Inc., Santa Cruz, CA, USA). Then, the slides were incubated with secondary antibody (goat anti-mouse conjugated to Alexa Fluor 488 or goat anti-rabbit conjugated to Alexa Fluor 566: 1:500, Molecular Probes, Eugene, OR, USA) for 1 h. Specimens were analyzed using an Olympus BX51 microscope and DP70 digital camera and software (Olympus, Tokyo, Japan).

### 4.9. Golgi-Cox Staining

Spinal cords were longitudinally dissected for about a two mm section and transferred to a vial containing solution provided by the FD Rapid GolgiStain kit (FD NeuroTechnologies, Inc., Ellicott City, MD, USA); we then followed the manufacturer’s protocol. The stained tissue was serially sectioned at 100-μm using a cryostat and then mounted onto gelatin-coated slides. The slides were analyzed by a blinded observer. Serial images were captured by a DCIT filter from ×1000 high magnification photographs and sharpened using Advanced SPOT software (Diagnostic Instruments, Sterling Heights, MI, USA). We imaged the dendrites of interneurons under a ×1000 oil immersion objective lens at a resolution of 0.027 × 0.027 × 0.3 μm. Approximately five to seven neurons were measured. The number of dendritic spines was counted manually by a trained observer who was blind to the experimental conditions, and normalized to the control for a 10-μm segment length. The spine density represents the number of spines/μm in all groups.

### 4.10. Statistics

All analyses were performed using the SPSS software (version 19.0, IBM, Chicago, IL, USA). Data collected from repeated experiments are presented as mean ± SEM. Statistical significance of data according to interventions were analyzed with One-way ANOVA. Differences were considered statistically significant when the *p*-value was <0.05.

## 5. Conclusions

In conclusion, the current study suggests that exogenous melatonin administration combined with physical exercise increases histological and behavioral recovery. Additionally, this dual treatment appears to increase nestin-positive eNSPCs, driving effective reconstructed neuronal differentiation. Our findings suggest that melatonin may have therapeutic potential because it contributes to activating adult neural stem cells; at this time, conducting exercise simultaneously may help to enhance the intrinsic potential for recovery from spinal cord injury.

## Figures and Tables

**Table 1.** Oligonucleotide primers used for RT-PCR.

Gene	Promer sequence (5′ to 3′)	Product length (bp)	Gen bank accession No.
*GFAP*	F: TGG CCA CCA GTA ACA TGC AA	134	NM_017009
	R: TCA AGT CAG CAA CGT GGA AG		
*MAP2*	F: CAA AGA GAA GGT GGC AAA GC	200	NM_013066
	R: GTG GGC AAG GGA TTT CTA CA		
	R: TCA AGC TGC AGC ATC CAT AC		
*Oct4*	F: GAG GGA TGG CAT ACT GTG GAC	272	NM_013633
	R: GGT GTA CCC CAA GGT GAT CC		
*GAPDH*	F: GTA TGA CTC CAC TCA CGG CAA A	100	BC094037
	R: GGT CTC GCT CCT GGA AGA TG		

## Abbreviation

BBB: Basso, Beattie, and Bresnahan
BrdU: bromodeoxyuridine
GFAP: glial fibrillary acidic protein
GM-SCF: granulocyte-macrophage colony stimulating factor
LIF: leukemia inhibitory factor
MAP2: microtubule-associated protein 2
NG2: neural-glial 2
eNSPCs: endogenous neural stem/progenitor cells
Oct4: octamer-binding transcription factor 4
SCI: spinal cord injury
